# Safety and immunogenicity of the third (booster) dose of inactivated and recombinant protein SARS-CoV-2 vaccine for patients with endocrine-related cancer

**DOI:** 10.3389/fpubh.2023.1086872

**Published:** 2023-02-02

**Authors:** Shanshan Han, Yuping Yang, Tingrui Wang, Rui Song, Daixing Hu, Mingli Peng, Zijing Lin, Qin Deng, Hong Ren, Jia Ming

**Affiliations:** ^1^Department of Breast and Thyroid Surgery, The Second Affiliated Hospital of Chongqing Medical University, Chongqing, China; ^2^Department of Infectious Diseases, The Second Affiliated Hospital, Chongqing Medical University, Chongqing, China

**Keywords:** SARS-CoV-2, COVID-19, vaccine, immunogenicity, endocrine-related cancer, breast cancer, thyroid cancer

## Abstract

**Background:**

Our study aimed to evaluate the safety and immunogenicity of the third (booster) dose of the COVID-19 vaccine for patients with endocrine-related cancers.

**Methods:**

This observational study involved 94 breast cancer patients, 92 thyroid cancer patients, and 123 healthy individuals who had received the third (booster) dose of the COVID-19 vaccine. Data on the adverse effects, serum anti-receptor binding domain (RBD)-immunoglobulin (Ig) G, and neutralizing antibodies (NAbs) were collected prospectively.

**Results:**

The serum anti-RBD-IgG and NAb titers were significantly lower for the patients with endocrine-related malignancies than for the healthy controls (3.01 [IQR: 1.11–6.70] vs. 4.19 [1.95–9.11], *p* = 0.001; 0.23 [0.11–0.52] vs. 0.41 [0.22–0.78], *p* = 0.001), and the seroconversion rates of anti-RBD-IgG and NAbs showed similar results. The serum antibody titers and seroconversion rates were significantly lower for patients aged ≥65 years with endocrine-related cancers, but there were no significant differences related to gender, vaccine type, or cancer type. Subgroup analysis showed that the antibody titers and seroconversion rates were significantly lower for patients with intermediate to advanced breast cancer, HR–/Her2+ breast cancer, and breast cancer undergoing treatment than for healthy controls. In contrast, breast cancer patients who completed their treatment and those who received endocrine therapy after completing their treatment were not significantly different from healthy controls. The NAbs titers and seroconversion rates were significantly lower for patients with primary thyroid cancer (0.19 [IQR: 0.10–0.46] vs. 0.41 [0.22–0.78], *p* = 0.003; 55.9 vs. 84.9%, *p* < 0.001); the seroconversion rates were significantly higher for the patients with combined Hashimoto's thyroiditis than for those without it. Multiple linear regression showed that patients aged ≥65 years who were receiving treatment were at risk of having lower antibody levels.

**Conclusion:**

The third (booster) dose of the COVID-19 vaccine is safe and well-tolerated. Our data support a third (booster) dose of the SARS-CoV-2 vaccine for breast and thyroid cancer patients. Breast cancer patients aged ≥65 years who are receiving treatment should be more protected, while thyroid cancer and breast cancer patients who have completed their treatment can be vaccinated like the general population.

## 1. Introduction

The coronavirus disease 2019 (COVID-19) pandemic caused by severe acute respiratory syndrome coronavirus 2 (SARS-CoV-2) is still spreading globally (1). More than 600 million confirmed cases and 6 million deaths have been reported as of August 2022, which impose a heavy burden on global public health and markedly affects social and economic development (2). To date, there are no antiviral drugs against SARS-CoV-2 (3), and the key to resistance against it is prevention. Therefore, vaccination against SARS-CoV-2 is the key to mitigating the COVID-19 pandemic (4). An extensive vaccination campaign has been underway globally since December 2020. Approximately 70% of the global population has received at least two vaccine doses (5). The COVID-19 vaccination is extensive globally; however, COVID-19 is still very prevalent. There are two main reasons for this: the COVID-19 vaccines have not yet been able to form an immune barrier, except in a few countries, and SARS-CoV-2 is constantly mutating (6). The decrease in the effectiveness of the COVID-19 vaccine over time cannot also be ignored (7). Therefore, vaccine booster immunization is imperative. As of August 2022, 820 million people in China had completed booster immunizations, of which more than 176 million people were older than 60 years (8).

The main causative strain of the current global outbreak of SARS-CoV-2 is Omicron. Full vaccination was effective, with real-world vaccine protection rates remaining above 50%, before the emergence of Omicron. However, it became inadequate after the emergence of Omicron. Studies have shown that booster vaccination is effective in slowing down the epidemic caused by Omicron, resulting in an average vaccine protection rate well above the 50% threshold. Booster vaccines against the original strain of the virus have also been reported to be still protective against the mutated strain. A third booster dose induces strong cellular and humoral immunity and, in consequence, reduces the risk of SARS-CoV-2 infection by attenuating disappearance neutralizing antibodies after two doses of vaccination (9–11), and no rare serious adverse reactions have been reported (10–12). A meta-analysis showed that the third dose of COVID-19 vaccination increased the mean IgG seroconversion rate from 39 to 66% while increasing the effective IgG concentration by ~69% (13). Another prospective study showed that 85% of 20 patients with initial seronegative solid tumors experienced seroconversion after the booster immunization (14), which implies that cancer patients who did not achieve seroconversion with the initial vaccination have the opportunity of protection from the third dose of the vaccine. Furthermore, Arbel et al. (15) found that mortality from COVID-19 was 90% lower for individuals who received the booster vaccine than for those who did not. Booster immunization has been effective for cancer patients; however, data on the immunogenicity and safety of the third dose of vaccines for cancer patients are scarce. All of the above studies suggest the effectiveness of booster vaccines for cancer patients.

Breast and thyroid cancers are the most common endocrine-related cancers and the first and ninth most prevalent malignancies globally, respectively (16). However, there are limited reports of studies on the safety and immunogenicity of the SARS-CoV-2 vaccine for patients with breast and thyroid cancers. One study found significantly lower receptor-binding domain (RBD)-immunoglobulin (Ig) G antibody titers than controls in a cohort of 201 breast and lung cancer patients who received two doses of COVID-19 vaccine (17). However, this study did not distinguish breast cancer from lung cancer and the findings were not fully and accurately representative of breast cancer patients. In another study, the SARS-CoV-2 vaccine was found to be safe in 115 patients with thyroid cancer who received at least one dose of the SARS-CoV-2 vaccine; however, the time to regression of SARS-CoV-2 IgG positivity was significantly shorter (18), and the changes in the SARS-CoV-2 IgG titers were not reported. Moreover, the current studies on the SARS-CoV-2 vaccination for patients with malignancies are mainly focused on two doses. Studies on the changes in the SARS-CoV-2 IgG titers in patients with malignancies after the third (booster) dose of the vaccine are lacking, and further investigation of the role of the booster vaccine in patients with breast or thyroid cancers is imperative.

This study recruited 94 breast cancer patients, 92 thyroid cancer patients, and 123 healthy controls, and the immunogenicity and safety of the third dose of the SARS-CoV-2 vaccine for breast cancer and thyroid cancer patients were assessed. In addition, relevant factors affecting the antibody titers in cancer patients were evaluated.

## 2. Materials and methods

### 2.1. Study design

Patients with pathologically confirmed breast and thyroid cancers between 2013 and 2022 in the Second Affiliated Hospital of Chongqing Medical University and healthy controls who were pathologically confirmed to have benign breast and thyroid tumors at the same hospital were recruited consecutively between June 2022 and August 2022.

The inclusion criteria were as follows: (i) completion of three doses (booster) of vaccination [currently, the vaccines requiring three doses in China are mainly classified as inactivated vaccines (CoronaVac and BBIBP-CorV) and recombinant protein vaccine (ZF2001)]; (ii) age of >18 years; and (iii) having only one type of malignancy. The exclusion criteria were as follows: (i) history of COVID-19 infection; (ii) pregnancy; (iii) autoimmune diseases other than Hashimoto's thyroiditis or ongoing immunosuppressive therapy for any reason; and (iv) malignancies.

**Figure 1:**
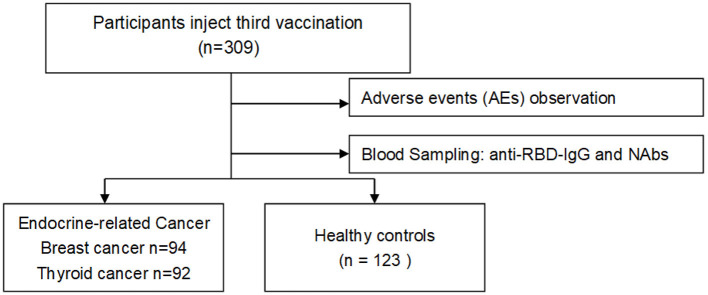
Flowchart of the study.

Blood specimens were first collected from participants who received three doses of vaccine, and serum anti-RBD-IgG and neutralizing antibody (NAb) titer levels were measured in all participants. Finally, a cross-sectional analysis was performed (Figure 1).

Adverse events (AEs) and basic patient information after the third vaccine dose were collected using questionnaires or *via* telephone at outpatient and inpatient units. The classification was based on the scale published by the State Drug Administration of China (2019 edition).

Treatment naïve (NT) individuals were considered to have never received treatment for a particular illness. Regarding active treatment (AT), active anticancer therapy was considered as chemotherapy, molecularly targeted therapy, or endocrine therapy within 6 months before or after vaccination; however, long endocrine therapy cycles for breast cancer patients were not classified as ongoing treatment. Previous treatment (PT) was considered as completed intensive anti-cancer treatment with an interval of at least 6 months at the time of the collection of the blood sample. Other clinical data were collected from our electronic medical database.

The study was approved by the Ethics Committee of the Second Affiliated Hospital of Chongqing Medical University, and the ethical guidelines of the Declaration of Helsinki were adhered to. Written informed consent was obtained from all participants.

### 2.2. SARS-CoV-2 antibody test

Plasma samples were collected for the detection of the IgG antibody against the RBD of the SARS-CoV-2 spike protein (anti-RBD-IgG) and NAbs with capture chemiluminescence immunoassays using MAGLUMITM X8 (Snibe, Shenzhen, China) according to the manufacturer's instructions. According to the kit specifications, anti-RBD-IgG tests have 100% sensitivity and 99.6% specificity, while NAb tests have 100% sensitivity and 100% specificity for the diagnosis of COVID-19. The cutoff value for NAbs was 0.15 ug/ml, while the cutoff value for anti-RBD-IgG was 1.0 AU/ml.

### 2.3. Statistical analysis

Appropriate methods were used for statistical analysis based on the type of data. Categorical variables were compared by Chi-square or Fisher's exact test. Continuous variables were compared with the Mann–Whitney *U* test (for unpaired data) or Wilcoxon test (for paired data) for two groups and the Kruskal–Wallis test for three groups. Antibody titer data is log-transformed and then compared. Using simple and multivariate regression analysis, clinical parameters associated with antibody titers were identified. The results of multiple comparisons were subjected to Bonferroni correction. Categorical variables were reported as numbers (%), continuous variables that conform to a normal distribution are reported as mean ± standard deviation, while those that do not are expressed as the median and interquartile range (IQR). *P*-values < 0.05 were considered to denote statistical significance. SPSS (IBM, version 26.0.0) was used for the statistical analysis, and GraphPad Prism (GraphPad Software Inc, 9.2.0) was used to create graphs.

## 3. Results

### 3.1. Participant characteristics

In total, 309 participants were enrolled in this study from June 2022 to August 2022; 186 were included in the endocrine-related cancer group, and 123 were included in the healthy control group. Of 309 participants, 284 were female, of these 172 (92.5%) were in the endocrine-related cancer group and 112 (91.1%) were in the healthy control group. The mean age of the patients was 48.6 ± 11.6 years for the endocrine-related cancer group and 46.1 ± 12.6 years for the healthy control group. The median number of days after vaccination was 217 days (IQR: 136–318 days) for the patients with endocrine-related cancers and 223 days (IQR: 166–301 days) for the healthy controls (Table 1). Of the 186 cancer patients, 51 (27.4%) received the Zhifei Longcom (China) vaccine, 135 (72.6%) received the Sinopharm vaccine, 94 (50.5%) had breast cancer, and 92 (49.5%) had thyroid cancer.

**Table 1:** Demographic characteristics of participants.

**Variables**	**Endocrine-related cancer (*n* = 186)**	**Healthy controls (*n* = 123)**	***P*-value**
Age (years)	48.6 ± 11.6	46.1 ± 12.6	0.072
Gender, female, *n* (%)	172 (92.5)	112 (91.1)	0.674
Days after 3rd dose vaccination, (days)	217 (136–318)	223 (166–301)	0.219
Vaccine type			0.229
Zhifei Longcom, China, *n* (%)	51 (27.4)	26 (21.1)	
Sinopharm vaccine, *n* (%)	135 (72.6)	97 (78.9)	
**Cancer type**			
Breast cancer, *n* (%)	94 (50.5)	/	/
Thyroid cancer, *n* (%)	92 (49.5)	/	/

### 3.2. Antibody responses to third dose of inactivated and recombinant protein SARS-CoV-2 vaccines

**Figure 2:**
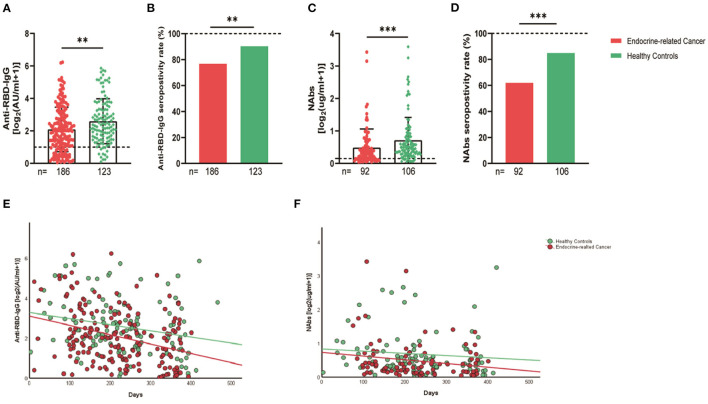
Antibody response to SARS-CoV-2 vaccine in patients with endocrine-related cancer and healthy controls. The titers and seropositivity rates of anti-RBD-IgG **(A, B)** and Nabs **(C, D)** in the sera of cancer patients and healthy individuals were measured. Anti-RBD-IgG **(E)** and NAbs **(F)** potencies were measured separately according to different days after immunization. Trend lines were generated using a single linear model fit. Error bars represent the median (IQR) (**P < 0.01, ***P < 0.001). Nabs, neutralizing antibodies; IQR, interquartile range; RBD, receptor binding domain.

**Figure 3:**
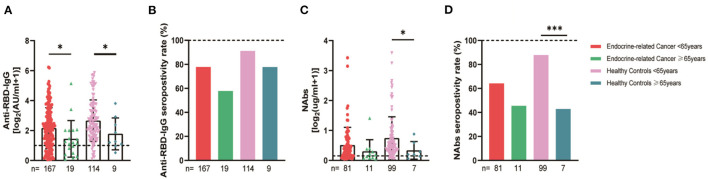
Antibody responses to inactivated SARS-CoV-2 vaccines in participants of different ages. Subgroup analysis of the titers and seropositivity rate of anti-receptor binding domain (RBD)-IgG **(A, B)** and NAbs **(C, D)** in participants according to age (*P < 0.05, ***P < 0.001). The horizontal dotted lines represent the limit of detection. The error bars represent the median (IQR). NAbs, neutralizing antibodies; IQR, interquartile range; RBD, receptor binding domain.

**Figure 4:**
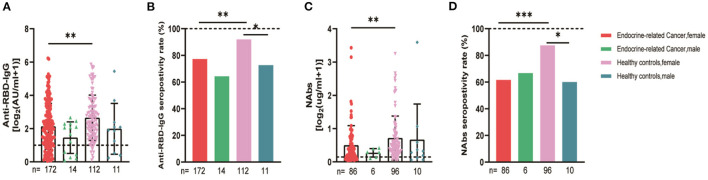
Antibody responses to inactivated SARS-CoV-2 vaccines in participants' gender. Subgroup analysis of the titers and seropositivity rate of anti-receptor binding domain (RBD)-IgG **(A, B)** and NAbs **(C, D)** in participants according to gender (*P < 0.05, **P < 0.01, ***P < 0.001). The horizontal dotted lines represent the limit of detection. The error bars represent the median (IQR). NAbs, neutralizing antibodies; IQR, interquartile range; RBD, receptor binding domain.

**Figure 5:**
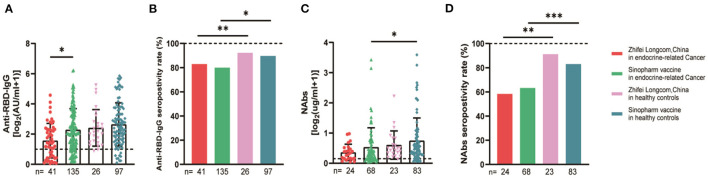
Antibody responses to SARS-CoV-2 vaccines in participants of different vaccine types. Subgroup analysis of the titers and seropositivity rate of anti-receptor binding domain (RBD)-IgG **(A, B)** and NAbs **(C, D)** in participants according to vaccine type. The horizontal dotted lines represent the limit of detection. The error bars represent the median (IQR) (*P < 0.05, **P < 0.01, ***P < 0.001). NAbs, neutralizing antibodies; IQR, interquartile range; RBD, receptor binding domain.

The serum anti-RBD-IgG titers and seroprevalence were significantly lower for the patients with endocrine-related cancer than for the healthy controls (3.01 [IQR: 1.11–6.70] vs. 4.19 [1.95–9.11], *p* = 0.001; 76.3 vs. 90.2%, *p* = 0.002). The NAb titers and seroconversion rates were also significantly lower (0.23 [0.11–0.52] vs. 0.41 [0.22–0.78], *p* = 0.001, 62.0% vs. 84.9, *p* < 0.001). The serum anti-RBD-IgG and NAb titers decreased more rapidly for patients with endocrine-related malignancies than for healthy participants (Figure 2). Subgroup analysis showed that the participants in both cancer and healthy control groups aged ≥65 years had lower anti-RBD-IgG antibody levels (1.28 [0.41–3.04] vs. 3.11 [1.11–7.53], *p* = 0.013; 1.38 [1.04–5.37] vs. 4.26 [2.15–10.38], *p* = 0.046) (Figure 3). The gender distribution, vaccine types, and antibody titers were significantly different for the patients with endocrine-related malignancies and healthy controls, and the antibody levels were significantly lower for the cancer group than for the healthy controls irrespective of gender or vaccine type (Figures 4, 5), which is consistent with the results of our overall analysis.

**Figure 6:**
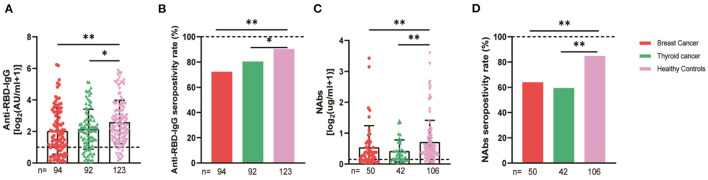
Antibody response to SARS-CoV-2 vaccine in patients with different cancer. The titers and seropositivity rates of anti-RBD-IgG **(A, B)** and NAbs **(C, D)** in the sera of cancer patients were measured. The horizontal dotted lines represent the limit of detection. The error bars represent the median (IQR) (*P < 0.05, **P < 0.01). NAbs, neutralizing antibodies; IQR, interquartile range; RBD, receptor binding domain.

We compared the breast and thyroid cancer patients separately with the healthy controls, and the serum anti-RBD-IgG, NAb titers, and seroconversion rates were significantly lower for the breast cancer group (2.36 [0.92–8.22] vs. 4.19 [1.95–9.11], *p* = 0.001, 72.3 vs. 90.2%, *p* = 0.001; 0.24 [0.10–0.55] vs. 0.41 [0.22–0.78], *p* = 0.007, 60 vs. 84.9%, *p* = 0.004). Similar results were obtained for the thyroid cancer group, with no significant differences in the breast and thyroid cancer groups (Figure 6).

**Figure 7:**
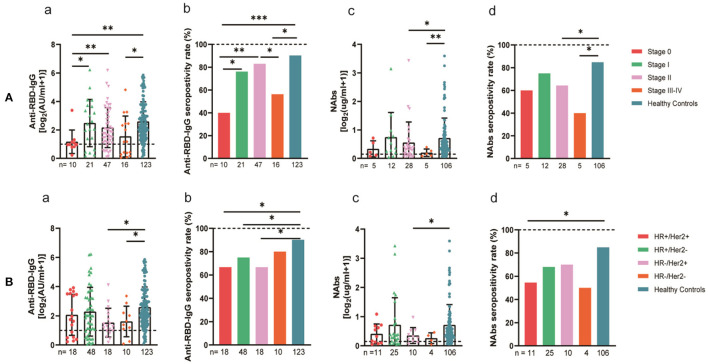
Antibody response to SARS-CoV-2 vaccine in breast cancer patients. **(A)** Antibody response to SARS-CoV-2 vaccine in breast cancer patients at the AJCC stages. **(B)** Antibody response to SARS-CoV-2 vaccine in breast cancer patients with different molecular typing. Subgroup analysis of the titers and seropositivity rate of anti-receptor binding domain (RBD)-IgG (a, b) and NAbs (c, d) in breast cancer participants. The horizontal dotted lines represent the limit of detection. The error bars represent the median (IQR) (*P < 0.05, **P < 0.01, ***P < 0.001). HR, hormone receptor; Her2, human epidermal growth factor receptor 2; NAbs, neutralizing antibodies; IQR, interquartile range; RBD, receptor binding domain.

**Figure 8:**
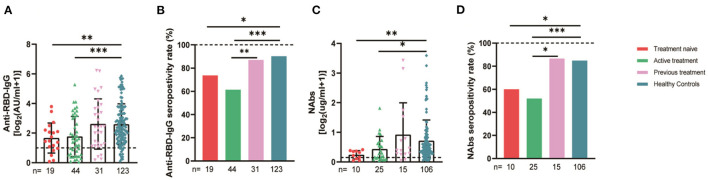
Antibody response to SARS-CoV-2 vaccine in breast cancer patients with different treatments status. Subgroup analysis of the titers and seropositivity rate of anti-receptor binding domain (RBD)-IgG **(A, B)** and NAbs **(C, D)** in participants according to different treatments. The horizontal dotted lines represent the limit of detection. The error bars represent the median. (IQR) (*P < 0.05, **P < 0.01, ***P < 0.001). NAbs, neutralizing antibodies; IQR, interquartile range; RBD, receptor binding domain. *Chemotherapy, molecularly targeted therapy, or radiotherapy within 6 months after or before vaccination was considered active anticancer therapy. *A person is considered to be treatment-naive if they have never undergone treatment for a particular illness.

**Figure 9:**
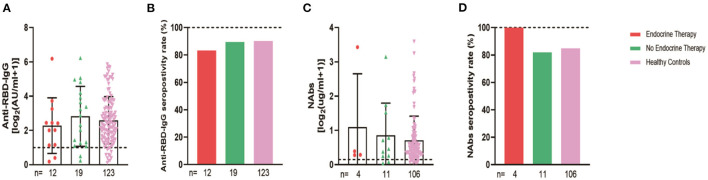
Antibody response to SARS-CoV-2 vaccine in breast cancer patients with or without Endocrine Therapy. Subgroup analysis of the titers and seropositivity rate of anti-receptor binding domain (RBD)-IgG **(A, B)** and NAbs **(C, D)** in participants according to different treatments. The horizontal dotted lines represent the limit of detection. The error bars represent the median (IQR). NAbs, neutralizing antibodies; IQR, interquartile range; RBD, receptor binding domain.

The 94 breast cancer patients were subgrouped according to their stages based on the AJCC 8th edition staging system; 10, stage 0; 21, stage I; 47, stage II; and 16, stages III-IV. The results showed that the anti-RBD-IgG antibody titers and seroconversion rates were significantly lower for the patients with stages III-IV than for the healthy controls (1.25 [0.23–4.44] vs. 4.19 [1.95–9.11], *p* = 0.046; 56.3 vs. 90.2%, *p* = 0.011); the NAb titers and seroconversion rates were also significantly lower (0.12 [0.06–0.26] vs. 0.41 [0.22–0.78], *p* = 0.008; 40.0 vs. 84.9%, *p* = 0.035) (Figure 7A). In addition, the seroconversion rate of the antibody titers was significantly lower for stage 0 patients than for stage I and stage II patients and healthy controls. We further analyzed the changes in antibody titers in patients with different molecular subtypes of breast cancer, including 18 cases of the HR+/Her2+ subtype, 48 cases of the HR+/Her2– subtype, 18 cases of the HR–/Her2+ subtype, and 10 cases of the HR–/Her2– subtype. The HR–/Her2+ anti-RBD-IgG antibody titers and seroconversion rates were significantly lower than those of the healthy controls (Figure 7B) (1.44 [IQR: 0.91–3.42] vs. 4.19 [1.95–9.11], *p* = 0.017; 66.7 vs. 90.2%, *p* = 0.013); except for studies with different staging and molecular subtypes. We also classified patients according to their treatment status at the time of blood sample collection into the NT, AT, and PT groups, with the highest proportion of patients in the active treatment group (41/94; 64.6%). The results showed significant reductions in the anti-RBD-IgG antibody titers and seroconversion rates for both the NT and AT groups relative to that for the healthy controls (NT: 1.75 [0.96–3.60] vs. 4.19 [1.95–9.11], *p* = 0.048; 73.7 vs. 90.2%, *p* = 0.039) (AT: 1.82 [0.45–7.39] vs. 4.19 [1.95–9.11], *p* = 0.003; 60.4 vs. 90.2%, *p* = 0.013). The results for the serum NAbs were consistent with the above; there was no significant difference between the PT group and healthy controls (Figure 8). In addition, the antibody levels of the patients receiving endocrine therapy at the end of treatment were also not different from those of the healthy controls (Figure 9).

**Figure 10:**
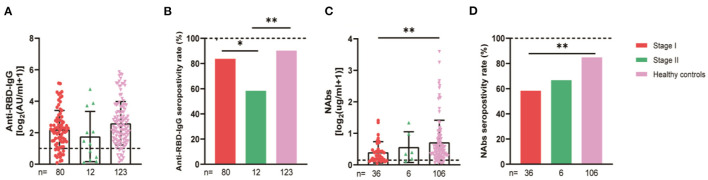
Antibody response to SARS-CoV-2 vaccine in thyroid cancer patients at the AJCC stages. Subgroup analysis of the titers and seropositivity rate of anti-receptor binding domain (RBD)-IgG **(A, B)** and NAbs **(C, D)** in participants according to different treatments. The horizontal dotted lines represent the limit of detection. The error bars represent the median (IQR) (*P < 0.05, **P < 0.01). NAbs, neutralizing antibodies; IQR, interquartile range; RBD, receptor binding domain.

**Figure 11:**
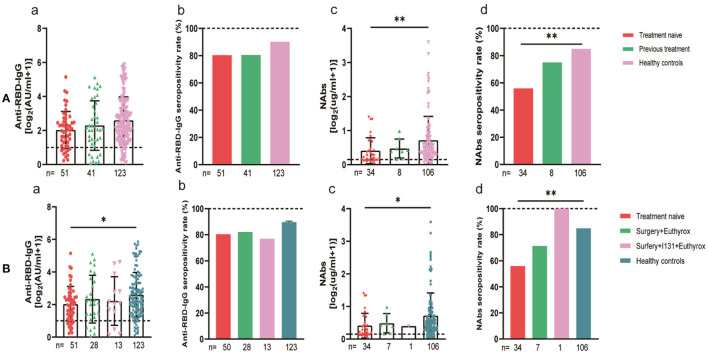
Antibody response to SARS-CoV-2 vaccine in thyroid cancer patients with different treatments. **(A)** Antibody responses to SARS-CoV-2 vaccines in thyroid cancer patients with or without treatment. **(B)** Antibody responses to SARS-CoV-2 vaccines in thyroid cancer patients with or without Surgery and (or) I131 treatment. Subgroup analysis of the titers and seropositivity rate of anti-receptor binding domain (RBD)-IgG (a, b) and NAbs (c, d) in participants according to different treatments. The horizontal dotted lines represent the limit of detection. The error bars represent the median (IQR) (*P < 0.05, **P < 0.01). NAbs, neutralizing antibodies; IQR, interquartile range; RBD, receptor binding domain.

For subgroup analysis, the thyroid cancer patients were grouped by their stages. The stages of the 92 thyroid cancer patients were as follows: 80 had stage I, 12 had stage II, and no patients had stages III–IV. The statistics showed that the seroconversion rate of the anti-RBD-IgG antibodies in stage II patients was significantly lower than that of the healthy control group (58.3 vs. 90.2%, *p* = 0.008), which was also significantly lower than that of the stage I patients (58.3 vs. 83.8%, *p* = 0.038) (Figure 10). The thyroid cancer patients were also stratified by their treatment statuses. There were no patients receiving treatment in this group, therefore, they were divided into 51 patients in the NT group and 41 patients in the PT group, which was further divided into Surgery + Euthyrox and Surgery + Euthyrox + I131 according to the specific treatment received. The results showed that the Nab titers and seroconversion rates were significantly lower for the NT group (0.19 [IQR: 0.10–0.46] vs. 0.41 [0.22–0.78], *p* = 0.003; 55.9 vs. 84.9%, *p* < 0.001), but they were not significantly different for the PT group and the healthy population (Figure 11). However, the antibody titers and seroconversion rates of the thyroid cancer patients who completed treatment were not significantly different from those of the healthy population (Figures 11A, B). Finally, the participants were grouped according to whether Hashimoto's thyroiditis was combined, and 29.3% (27/92) of the patients in the cancer group had HT and 8.1% (10/123) of the healthy control group had HT, and it was found that the seroconversion rate of the subjects with HT was significantly higher than that of subjects without HT, although there was no significant difference between antibody titers (Supplementary Figure 1).

Next, we performed linear regression to determine the factors affecting anti-RBD-IgG titers. Simple linear regression showed that interval, age of ≥65 years, HR–/HER2+, treatment-naïvity, and AT were associated with low antibody titers in breast cancer patients (Table 2). Multiple linear regression showed that age of ≥65 years and AT were associated with low antibody titers in breast cancer patients (Table 2). Simple and multiple linear regression analyses of NAbs identified AT as a risk factor for reduced antibody titers in breast cancer patients (Supplementary Table 1). Both simple and multiple linear regression in patients with thyroid cancer found interval time as the only factor associated with reduced antibody titers (Table 3). Different results were found for NAbs (Supplementary Table 1). During the follow-up, all participants in this study were free of COVID-19 infection. Of course, this may be due to our country's epidemic prevention policy.

**Table 2:** Simple and multiple regression analyses to identify risk factors of lower anti-RBD titers in breast cancer patients.

**Variables**	**Simple linear regression β value (95% CI)**	***P*-value**	**Multiple linear regression β value (95% CI)**	***P*-value**
Time^a^	−0.038 (−0.062, −0.013)	0.003	−0.020 (−0.059, 0.019)	0.306
Age	−0.243 (−0.483, −0.004)	0.047	−0.322 (−0.575, −0.068)	0.013
**Vaccine type**				
Zhifei Longcom, China	Reference			
Sinopharm vaccine	5.342 (−0.075, 10.759)	0.053	–	–
**Stage**				
0	Reference			
I	8.134 (−1.090, 17.358)	0.083	–	–
II	5.193 (−3.167, 13.554)	0.220	–	–
III + IV	2.225 (−7.453, 11.903)	0.649	–	–
**Molecular type**				
HR+/Her2+	Reference			
HR+/Her2–	0.166 (−2.582, 10.591)	0.230	–	–
HR–/Her2+	−0.08 (−10.398, 5.489)	0.541	–	–
HR–/Her2–	−0.061 (−11.786, 7.011)	0.615	–	–
**Anticancer therapy status**				
Previous treatment	Reference			
Treatment naïve	−0.284 (−15.342, −1.770)	0.014	−0.241 (−14.798, 0.316)	0.060
Active treatment	−0.293 (−12.536, −1.640)	0.011	−0.255 (−12.304, −0.032)	0.049
**Endocrine therapy**				
No endocrine therapy	Reference			
Active endocrine therapy	−0.090 (−17.524, 10.807)	0.631	–	–

^a^Day after 3rd dose vaccination; RBD, receptor binding domain; NAbs, neutralizing antibodies.

**Table 3:** Simple and multiple regression analyses to identify risk factors of lower anti-RBD titers in thyroid cancer patients.

**Variables**	**Simple linear regression β value (95% CI)**	***P*-value**	**Multiple linear regression β value (95% CI)**	***P*-value**
Time^a^	−0.018 (−0.033, −0.002)	0.024	−0.018 (−0.033, −0.002)	0.024
**Sex**				
Male	Reference			
Female	4.032 (−0.102, 8.166)	0.056	–	–
**Age**	0.03 (−0.095, 0.156)	0.633	–	–
**Vaccine type**				
Zhifei Longcom, China	Reference			
Sinopharm vaccine	2.824 (−0.576, 6.224)	0.102	–	–
**Stage**				
I	Reference			
II	−0.23 (−5.001, 3.996)	0.825	–	–
**Hashimoto's thyroiditis**				
Thyroid cancer	Reference			
Thyroid cancer with HT^b^	2.667 (−0.614, 5.948)	0.11	–	–
**Treatment**				
Treatment native	Reference			
Previous treatment	2.457 (−0.548, 5.462)	0.108	–	–
**Treatment method**				
Surgery	Reference			
Surgery + I131	1.462 (−2.746, 5.670)	0.492	–	–

^a^Day after 3rd dose vaccination;

^b^Hashimoto's thyroiditis. RBD, receptor binding domain; NAbs, neutralizing antibodies.

### 3.3. Safety of the inactivated third dose of SARS-CoV-2 vaccines

As shown in Table 4, no any severe AEs were observed during the follow-up period. There were no significant differences in the overall incidence of AEs among the breast and thyroid cancer patients and the healthy population (8.1% for the healthy group and 11.7% for the breast cancer group, *p* = 0.129; 16.3% for the thyroid cancer group, *p* = 0.085). The local adverse reactions in cancer patients included injection site pain (7.4% for breast cancer; 15.2% for thyroid cancer), swelling (2.1% for breast cancer; 5.4% for thyroid cancer), redness (1.1% for breast cancer; 2.1% for thyroid cancer), and pruritus (1.1% for thyroid cancer). Only injection site pain was reported for the healthy controls. Patients in the thyroid cancer group were more likely to have localized pain than the healthy controls (5.7% for the healthy group and 15.2% for the thyroid cancer group, *p* = 0.035); the breast cancer and healthy control groups showed no difference in the incidence of localized pain. Systemic AEs were less frequent in cancer and healthy control groups, with dizziness and fatigue being the most common systemic AEs for all the participants. There were no significant differences in the incidence of systemic AEs in the thyroid and breast cancer and healthy control groups.

**Table 4:** Adverse events of COVID-19 vaccination in enrolled participants.

**AES within 7 days**	**Thyroid cancer (*n* = 92)**	**Breast cancer (*n* = 94)**	**Healthy controls (*n* = 123)**	***P*-Value**
Overall AES	15 (16.3%)	11 (11.7%)	10 (8.1%)	0.085^a^
				0.129^b^
**Local AES**				
Pain	14 (15.2%)	7 (7.4%)	7 (5.7%)	0.035^a^
				0.591^b^
Swelling	5 (5.4%)	2 (2.1%)	0	0.014^a^
				0.185^b^
Redness	2 (2.2%)	1 (1.1%)	0	0.183^a^
				0.431^b^
Itch	1 (1.1%)	0	0	0.429^a^
Induration	0	0	0	–
**Systemic AES**				
Muscle pain	0	1 (1.1%)	0	0.431^b^
Pruritus	0	0	0	–
Rash	0	0	1 (0.8%)	0.385^a^
Fatigue	–	1 (1.1%)	1 (0.8%)	0.385^a^
				0.730^b^
Drowsiness	0	0	0	–
Dizziness	1 (1.1%)	1 (1.1%)	1 (0.8%)	0.737^a^
				0.730^b^
Headache	0	0	0	–
Rhinorrhea	0	0	0	–
Laryngeal pain	0	0	0	–
Fever	0	1 (1.1%)	0	0.431^b^
Chill	0	0	0	–
Cough	0	0	0	–
Inappetence	0	0	0	–
Abdominal Pain	0	0	0	–
Abdominal Distension	0	0	0	–
Diarrhea	0	0	0	–
Hepatalgia	0	0	0	–
Nausea	0	0	0	–
Chest Distress	0	0	0	–
Constipation	0	0	0	–

^a^The Chi-square test was done between thyroid cancer and healthy control group.

^b^The Chi-square test was done between breast cancer and healthy control group.

## 4. Discussion

Real-world data show that a complete three-dose (including booster) SARS-CoV-2 vaccination delays the outbreak dominated by the Omicron variant while improving the disappearance of neutralizing antibodies and reducing the SARS-CoV-2 infection rates after the second dose. The third dose (booster) is safe and well-tolerated in healthy populations, but data from studies of cancer populations are still scarce.

In this article, we present the results of a cross-sectional study that investigated the safety and immunogenicity of the inactivated and recombinant protein SARS-CoV-2 vaccine booster for patients with endocrine-related malignancies (breast and thyroid) and healthy controls. We also evaluated factors affecting the serum antibody titers in patients with breast and thyroid cancers.

Previous studies have reported that the poor immune effect of the start-up dose of the COVID-19 vaccine in patients with solid cancers is compensated for by a subsequent vaccination, with seroprevalence reported to range from 75 to 95% throughout the study (19, 20). However, antibody titers and seroprevalence were lower in patients with solid tumors than in the healthy population (21–23). In this study, we found that serum anti-RBG-IgG and NAb titer levels were significantly lower for the breast and thyroid cancer patients than for normal healthy controls even with the booster injection, in addition to the lower RBG-IgG and NAb seroconversion rates. These results suggest that cancer patients are less immunogenic to SARS-CoV-2 vaccine booster shots. This may be due to the impaired humoral response to the new crown pneumonia vaccine in cancer patients (24, 25). Although impaired immune responses to vaccines in cancer patients are attributed to advanced disease and underlying disease-related immunosuppression, treatment-inducing factors, especially related treatment regimens, the timing of therapy, and concomitant vaccination, play a role in several cases (26).

The subgroup analysis for endocrine-related cancer showed that antibody titers and seroconversion rates were significantly lower for older people aged ≥65 years than for healthy controls. Studies have also reported a significant decrease in serum antibody titers after the vaccination of patients aged ≥65 years (27, 28). Older patients' immune systems are usually weaker, and their B and T cells are less responsive to external stimuli (29). In addition, we found no significant difference in immunogenicity between participants receiving inactivated or recombinant protein vaccines in the cancer or healthy control group.

The subgroup comparison of breast cancer patients showed lower antibody titers in patients with stages 0 and III–IV and the HR–/Her2+ subtype and those who were NT or receiving AT. The high proportion of patients (20%) aged 65 or more years with stage 0 cancer in our study may explain the lower antibody levels in these patients, and the presence of distant metastases in patients with intermediate to advanced disease may have further aggravated the immune damage and the resultant lower antibody levels. Some studies have found that HER2+ breast cancer usually has higher levels of stromal tumor-infiltrating lymphocytes (TIL) than HR+/HER2– breast cancer, implying that HER2+ disease is usually more immunogenic. Secondly, not all HER2+ tumors are immunogenic, and specific molecular HER2+ subgroups (e.g., HER2 enriched) are more immunogenic than others (e.g., luminal A/B) (30). Our study found that HER2+ breast cancer patients had lower antibody titers and poorer immunogenicity. According to the guidelines, patients with this breast cancer phenotype usually require long-term chemotherapy and targeted therapy, and this may have accounted for the above finding.

In our study, we did not determine whether chemotherapy and targeted therapy were administered, because the main anti-cancer treatment strategies for breast cancer, except endocrine therapy which lasts for 5–10 years, are focused on surgery, chemotherapy, targeted therapy, and radiotherapy within 6 months to a year of diagnosis and require a combination or alternation of treatment modalities. This makes it difficult to determine the effect of a particular treatment modality on patients. Due to the small sample of our study, we only grouped the patients based on their treatment status at the time of blood sample collection and not their specific treatments. Across the different treatment states, we found significantly lower serum antibody levels and seroconversion rates for the patients receiving the initial and ongoing treatment than for the healthy controls, while the antibody levels of the treated patients did not differ from those of the healthy controls. The lower levels of antibodies in the primary patients, after excluding the interference of treatment, confirm the impaired immune function of cancer patients, while the lower levels of the antibodies in the patients undergoing treatment suggest a suppressive effect of anti-cancer treatment on the immune system of the patients. We also found no significant differences between the serum antibody titers and seroconversion rates of the breast cancer patients who had completed treatment, those who received endocrine therapy after completing treatment, and the control group. We can infer that the tumor carriage status and the suppression of autoimmunity by ongoing treatment were temporary, and the serological response to vaccination after the discontinuation of intensive anticancer treatment was comparable to that of the healthy population. In addition, we found no effect of endocrine therapy on immune function in breast cancer patients after the end of intensive anti-cancer treatment.

Multiple linear regression showed that age of ≥65 years and ongoing treatment were risk factors for reduced antibody titers in breast cancer patients. The immune function of older patients was relatively weakened, and patients undergoing treatment had a poor response to vaccination due to changes in immune function caused by the tumor, on the one hand, and the use of therapies that strongly suppress the immune function of the body, such as chemotherapy, on the other hand.

Subgroup analysis of the thyroid cancer patients showed a higher seroconversion rate for participants with Hashimoto's thyroiditis (HT). HT is the most common autoimmune disease globally, and it is characterized by chronic inflammation, increased circulating concentrations of autoantibodies against thyroid peroxidase and thyroglobulin, and tertiary lymphoid follicle development. CD4+ T cells are overstimulated, which leads to the overstimulation and production of B and plasma cells (31). It has also been found that the increase in plasma cells in HT (32) may be responsible for the increased seroconversion rate. However, studies related to HT and the COVID-19 vaccine are lacking in the literature, and this necessitates further exploration. We also found that NAb titers and seroprevalence rates were significantly lower for untreated thyroid cancer patients but those for the treated thyroid cancer patients and the healthy population were not significantly different. This may indicate that the immune function of treated thyroid cancer patients does not differ from that of the healthy group and can be managed as for the healthy group. The difference in treatment modalities did not affect the immunogenicity of thyroid cancer patients. Multiple linear regression showed no other risk factors for lower antibody titers in thyroid cancer.

Based on the World Health Organization's guidelines, AEs can be classified according to their seriousness into mild-to-moderate and severe events. Mild-to-moderate side effects include pain at the injection site, fever, fatigue, headache, muscle pain, chills, diarrhea, and so on, which are normal and not a cause for alarm: they are signs that the body's immune system is responding to the vaccine, specifically the antigen (a substance that triggers an immune response), and is gearing up to fight the virus. Previous studies on the safety of the COVID-19 vaccine in cancer patients have reported no severe adverse reactions after vaccination (33). In the present study, the booster vaccine was also generally safe and well-tolerated in breast and thyroid cancer patients. However, our study suggested a higher risk of local AEs in patients with thyroid cancer. Recall observer bias may have affected this result, given the long interval between the booster injections and the investigations. Furthermore, no severe adverse reactions, such as thromboembolic events, myocardial infarction, convulsions, erythema multiforme, and Stevens-Johnson syndrome, in all populations have been reported by previous studies. The most commonly reported adverse reaction is local pain. Even though 41 breast cancer patients were receiving active anti-cancer therapy (chemotherapy, molecular targeted therapy, and radiotherapy), no severe adverse reactions were observed, which further demonstrated the safety of the booster vaccine.

Our current study has several limitations. First, we only examined the levels of RBD-IgG antibodies and neutralizing antibodies and not the level of SARS-CoV-2-specific memory B-cell response. Second, we did not examine the levels of T cell response, which is essential for adaptive immune responses against COVID-19, and T cells may be better associated with long-term immune memory and prevention of severe disease than somatic responses (34). Third, we did not measure the serum levels of antibodies against the Omicron variant of the coronavirus in the study participants. Fourth, we collected information on adverse events at the time of enrolment, rather than after vaccination, which is not sufficiently accurate and some adverse events may be overlooked or forgotten by participants due to memory errors. Fifth, this was a cross-sectional study, and there was no long-term follow-up to observe antibody changes to determine the duration of serologic response. Finally, our study may have been influenced by uncontrollable factors, such as population, geography, and access bias, because it was conducted in a single center.

## 5. Conclusion

The third dose of (booster) inactivated and recombinant protein SARS-CoV-2 vaccines are safe and well-tolerated in patients with endocrine-related cancers. The antibody response after the third dose (booster) is weaker in cancer patients, especially breast cancer patients aged ≥65 years old, those with intermediate and advanced stages, those with the HR–/Her2+, and those receiving anti-cancer therapy. These patients require more protection, and cancer patients who have completed treatment can be treated like healthy individuals for epidemic management.

## Data availability statement

The raw data supporting the conclusions of this article will be made available by the authors, without undue reservation.

## Ethics statement

The studies involving human participants were reviewed and approved by the Ethics Committee of the Second Affiliated Hospital of Chongqing Medical University. The patients/participants provided their written informed consent to participate in this study.

## Author contributions

SH and YY were responsible for the draft writing, data collection, and contributed equally to this study. TW, RS, DH, MP, ZL, and QD were responsible for the data collection, data analysis, and follow-up of the patients. HR and JM were responsible for the design and supervision of this study. All authors contributed to the article and approved the submitted version.
