# Supplementary material for: Safety and immunogenicity of the third (booster) dose of inactivated and recombinant protein SARS-CoV-2 vaccine for patients with endocrine-related cancer
Source: Front Public Health. 2023 Feb 2;11:1086872. doi: 10.3389/fpubh.2023.1086872 (PMC9932592; doi:10.3389/fpubh.2023.1086872)
Supplement: Supplementary file 1 [file Table_1.DOCX]

**Supplementary Table 1**   Simple and multiple regression analyses to identify risk factors for lower NAb titers in breast cancer patients.

| **Variables** | **Simple linear regression β value (95% CI)** | ***P*-value** | **Multiple linear regression β value (95% CI)** | ***P*-value** |
| --- | --- | --- | --- | --- |
| **Time ^a^** | -0.005 (-0.010, 0.000) | 0.062 | - | - |
| **Age** | -0.027 (-0.076, 0.022) | 0.266 | - | - |
| **Vaccine type** |  |  |  |  |
| Zhifei Longcom, China | Reference |  |  |  |
| Sinopharm vaccine | 0.621 (-0.516, 1.759) | 0.277 | - | - |
| **Stage** |  |  |  |  |
| 0 | Reference |  |  |  |
| I | 0.842 (-1.072, 2.757) | 0.380 | - | - |
| II | 0.501 (-1.245, 2.247) | 0.566 | - | - |
| III+IV | -0.128 (-2.402, 2.146) | 0.910 | - | - |
| **Molecular type** |  |  |  |  |
| HR+/Her2+ | Reference |  |  |  |
| HR+/Her2- | 0.237 (-0.453, 2.104) | 0.200 | - | - |
| HR-/Her2+ | -0.015 (-1.608, 1.480) | 0.934 | - | - |
| HR-/Her2- | -0.024 (-2.219, 1.907) | 0.880 | - | - |
| **Anticancer therapy status** | |  |  |  |
| Previous treatment | Reference |  |  |  |
| Treatment naïve | -0.342 (-2.867, -0.107) | 0.035 | -0341 (-0.296, 0.026) | 0.054 |
| Active treatment | -0.361 (-2.360, -0.153) | 0.027 | -0.390 (-2.659, -0.053) | 0.042 |
| **Endocrine therapy** |  |  |  |  |
| No endocrine therapy | Reference |  |  |  |
| Active endocrine therapy | 0.199 (-2.543, 5.141) | 0.730 | - | - |

^a^ Day after 3rd dose vaccination; RBD = receptor binding domain; Nabs = neutralizing antibodies
