# Supplementary material for: Safety and immunogenicity of the third (booster) dose of inactivated and recombinant protein SARS-CoV-2 vaccine for patients with endocrine-related cancer
Source: Front Public Health. 2023 Feb 2;11:1086872. doi: 10.3389/fpubh.2023.1086872 (PMC9932592; doi:10.3389/fpubh.2023.1086872)
Supplement: Supplementary file 2 [file Table_2.DOCX]

**Supplementary Table 2**  Simple and multiple regression analyses to identify risk factors for lower NAb titers in thyroid cancer patients.

| **Variables** | **Simple linear regression β value (95% CI)** | **P-value** | **Multiple linear regression β value (95% CI)** | **P-value** |
| --- | --- | --- | --- | --- |
| **Time ^a^** | 0.001 (0.000, 0.003) | 0.102 | - | - |
| **Sex** |  |  |  |  |
| Male | reference |  |  |  |
| Female | 0.199 (-0.166, 0.565) | 0.277 | - | - |
| **Age** | 0.004 (-0.008, 0.015) | 0.519 | - | - |
| **Vaccine type** |  |  |  |  |
| Zhifei Longcom, China | reference |  |  |  |
| Sinopharm vaccine | 0.085 (-0.209, 0.379) | 0.562 | - | - |
| **Stage** |  |  |  |  |
| I | Reference |  |  |  |
| II | 0.163 (-0.155, 0.485) | 0.303 | - | - |
| **Hashimoto’s thyroiditis** |  |  |  |  |
| thyroid cancer | reference |  |  |  |
| thyroid cancer with HT^b^ | 0.068 (-0.247, 0.384) | 0.663 | - | - |
| **Treatment** |  |  |  |  |
| Treatment native | reference |  |  |  |
| Previous treatment | 0.032 (-0.298, 0.362) | 0.845 | - | - |
| **Treatment method** |  |  |  |  |
| Surgery | reference |  |  |  |
| Surgery+I131 | 0.563 (-0.019, 1.145) | 0.058 | - | - |

^a^ Day after 3rd dose vaccination; ^b^ Hashimoto’s thyroiditis. RBD = receptor binding domain; Nabs = neutralizing antibodies
