# Supplementary figures and images for: Safety and immunogenicity of the third (booster) dose of inactivated and recombinant protein SARS-CoV-2 vaccine for patients with endocrine-related cancer
Source: Front Public Health. 2023 Feb 2;11:1086872. doi: 10.3389/fpubh.2023.1086872 (PMC9932592; doi:10.3389/fpubh.2023.1086872)

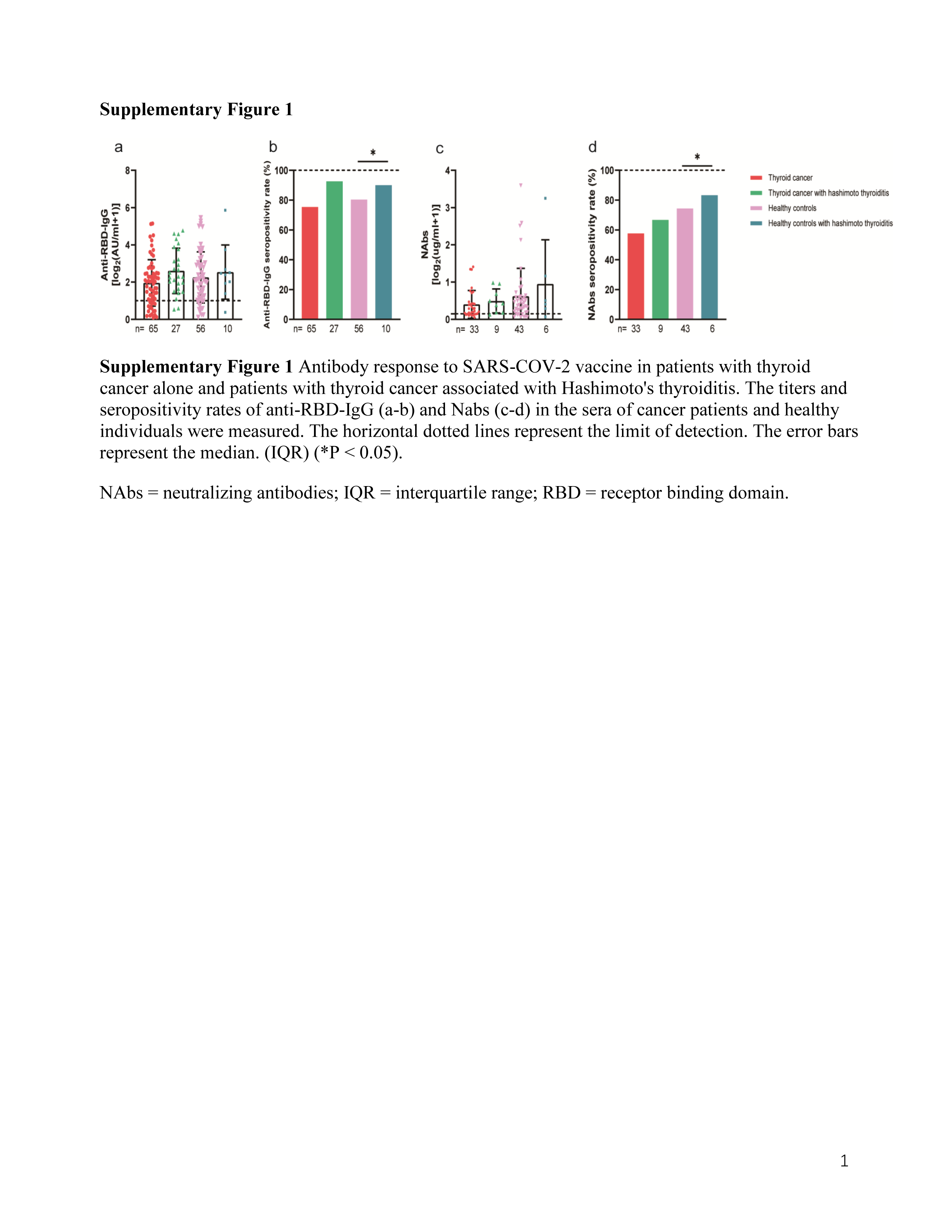

Supplement: Supplementary file 3 [file Image_1.TIF]
